# Supplementary material for: Directed self-assembly of viologen-based 2D semiconductors with intrinsic UV–SWIR photoresponse after photo/thermo activation
Source: Nat Commun. 2020 Mar 4;11:1179. doi: 10.1038/s41467-020-14986-7 (PMC7055315; doi:10.1038/s41467-020-14986-7)
Supplement: Supplementary file 1 — Supplementary Information [file 41467_2020_14986_MOESM1_ESM.pdf]

Supplementary Information

Directed Self-Assembly of Viologen-based 2D Semiconductors with Intrinsic UV–SWIR Photoresponse after Photo/thermo Activation

Xiao-Qing Yu, [†,§] Cai Sun, [†] Bin-Wen Liu, [†] Ming-Sheng Wang[†]* & Guo-Cong Guo[†]

[†]*State Key Laboratory of Structural Chemistry, Fujian Institute of Research on the Structure of Matter, Chinese Academy of Sciences (CAS), 155 Yangqiao Road West, Fuzhou, Fujian 350002, China*

[§]*University of Chinese Academy of Sciences, No.19A Yuquan Road, Beijing 100049, China*

*email: mswang@fjirsm.ac.cn (M.-S. Wang)

Index

1 Supplementary Figures. 2

2 Supplementary Tables. 11

3 Supplementary Note 1: 12

Measurement of the relative spin density. 12

1 Supplementary Figures.

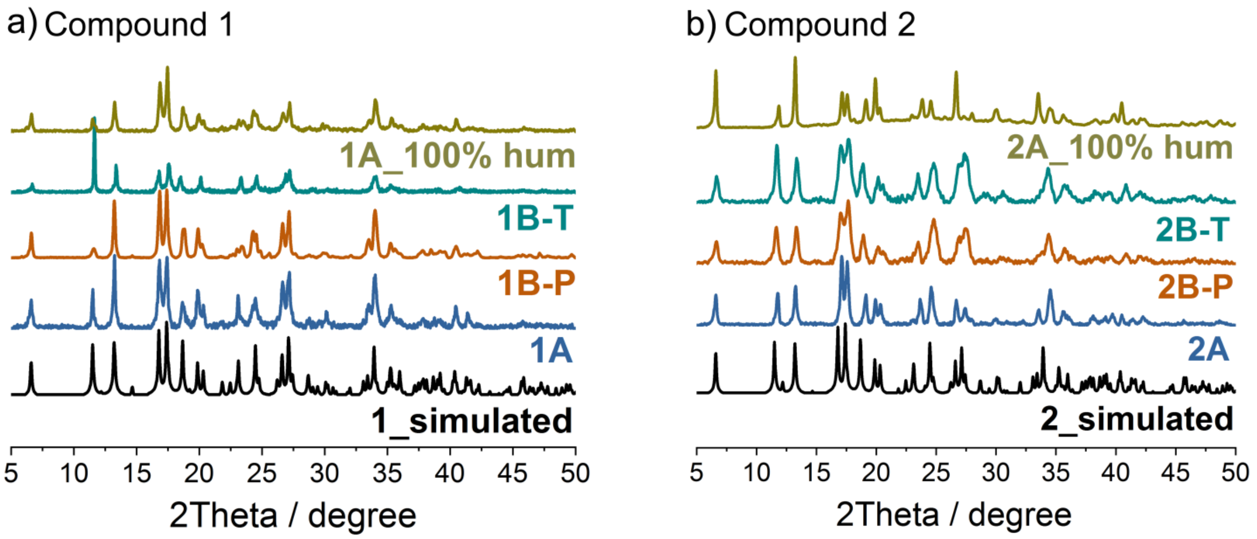

Supplementary Fig. 1 **PXRD patterns of 1 and 2**. a) **1_simulated**, **1A**, **1B-P**, **1B-T**, **1A_100% hum**; b) **2_simulated**, **2A**, **2B-P**, **2B-T**, **2A_100% hum.** Note: **1_simulated**/**2_simulated** refer to simulated PXRD curves that were simulated using the single-crystal X-ray diffraction data. **1A**/**2A** are as-prepared crystalline samples; **1B-P**/**2B-P** are samples that were irradiated by an Xe lamp for 70 min; **1B-T**/**2B-T** are samples that were thermally annealed at 180 °C for 150 min; **1A_100% hum**/**2A_100% hum** are samples that were placed in the dark under the 100% relative humidity for 12 h.

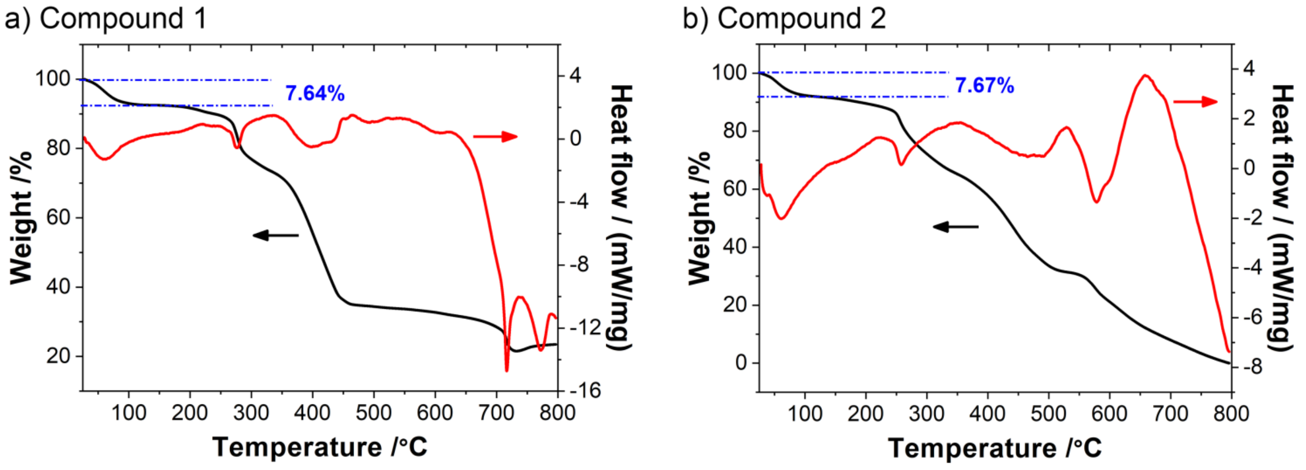

Supplementary Fig. 2 **Thermogravimetric analysis**. The data for **1A** (a) and **2A** (b) measured in nitrogen atmosphere with a ramp rate of 5 °C min^{-1}.

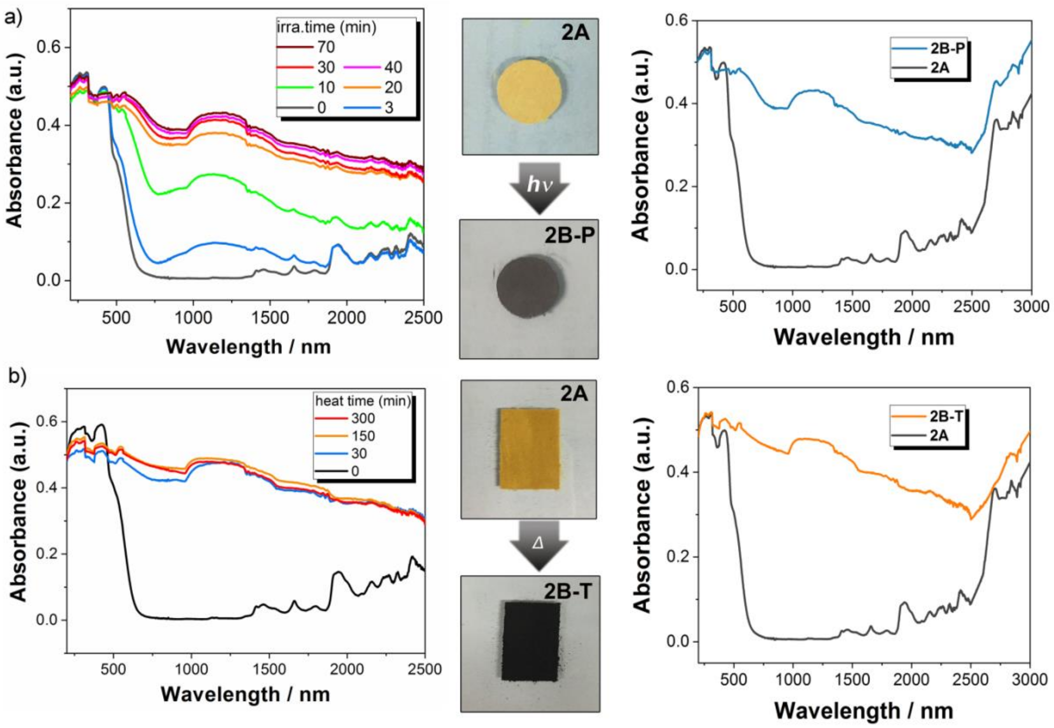

Supplementary Fig. 3 Electron absorption spectra and color change of 2. Time-dependent electron absorption spectra measured in the diffuse reflectance mode, color change, and combined UV/Visible/NIR spectra (200–2500 nm) and IR spectra (2500–3000 nm) of **2** upon irradiation at room temperature (a) or thermal annealing at 180 °C (b).

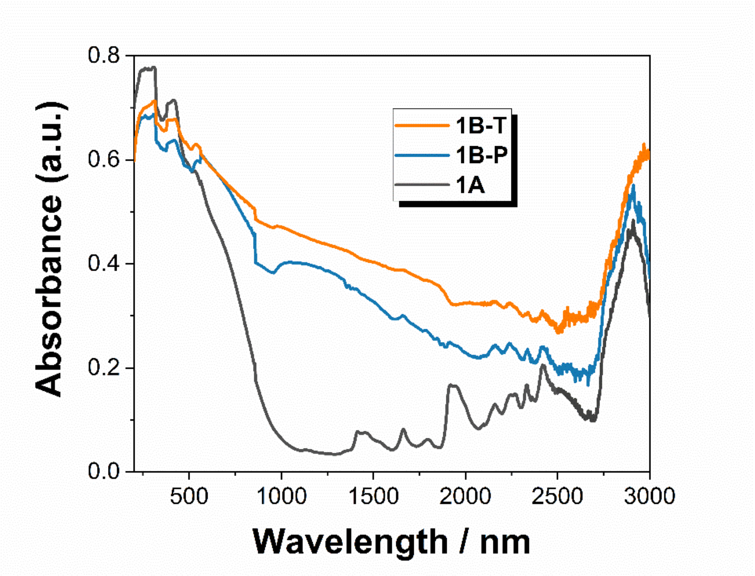

Supplementary Fig. 4 Multispectral absorption of 1A, **1B-P and 1B-T**. Combined UV/Visible/NIR spectra (200–2500 nm) and IR spectra (2500–3000 nm) for **1A**, **1B-P**, and **1B-T**.

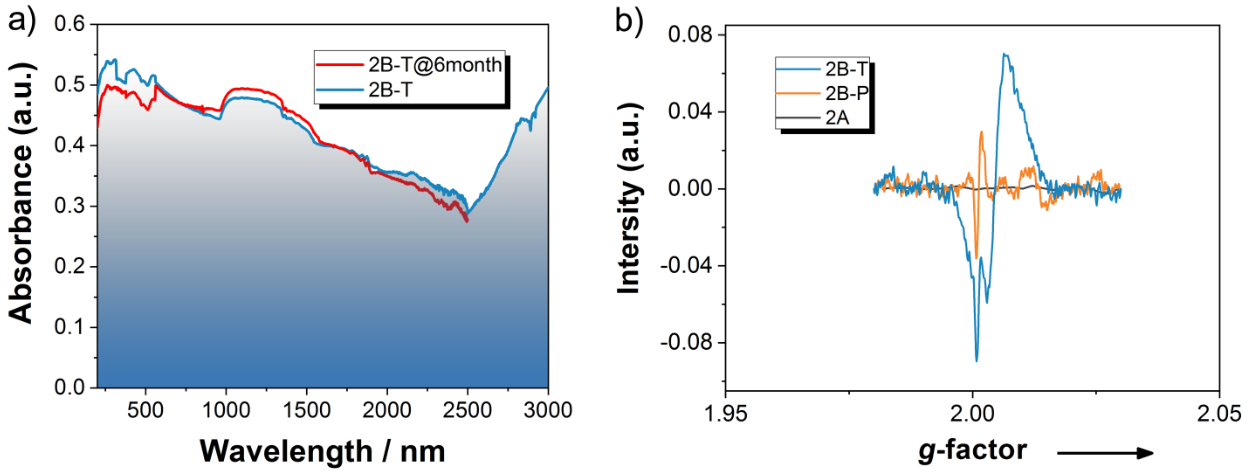

Supplementary Fig. 5 Air-stability and ESR test. a) Stability test for the thermo-induced sample **2B-T** monitored using UV/Visible/NIR spectra (200–2500 nm) and IR spectra (2500–3000 nm). The sample was placed in air in the dark at room temperature for 6 month. The minor difference between **2B-T** and **2B-T@6month** was attributed to systematic errors caused by instrumental instability or shift of the sample holder. b) ESR patterns for **2A**, **2B-P** and **2B-T**. Note: **2A** is as-prepared crystalline sample; **2B-P** is sample that was irradiated by an Xe lamp for 70 min; **2B-T** is sample that was thermally annealed at 180 °C for 150 min.

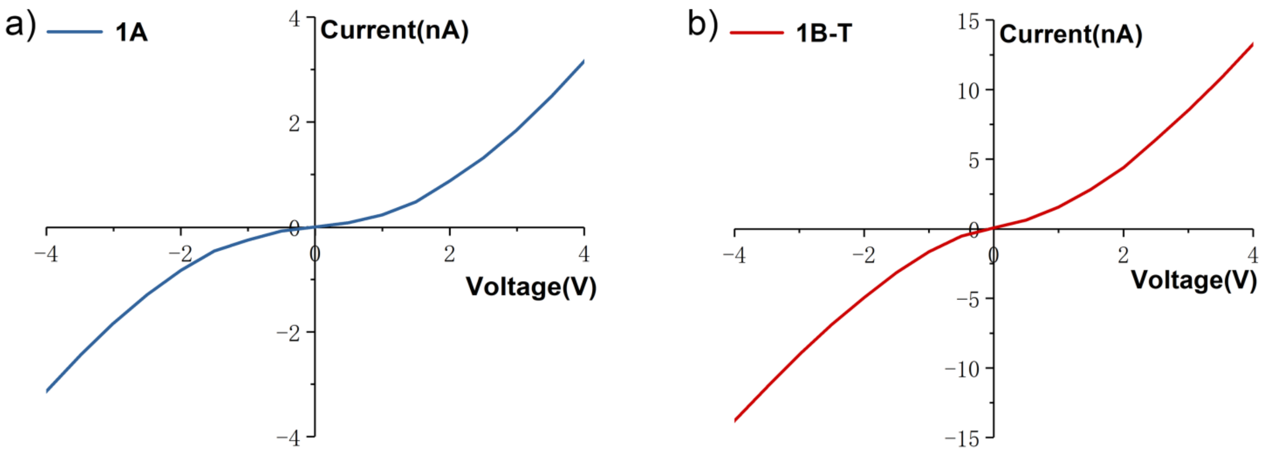

Supplementary Fig. 6 Current–voltage (*I*–*V*) plots. *I-V* plots featured Ohmic connect at 298 K in air for **1A** (a) and **1B-T** (b).

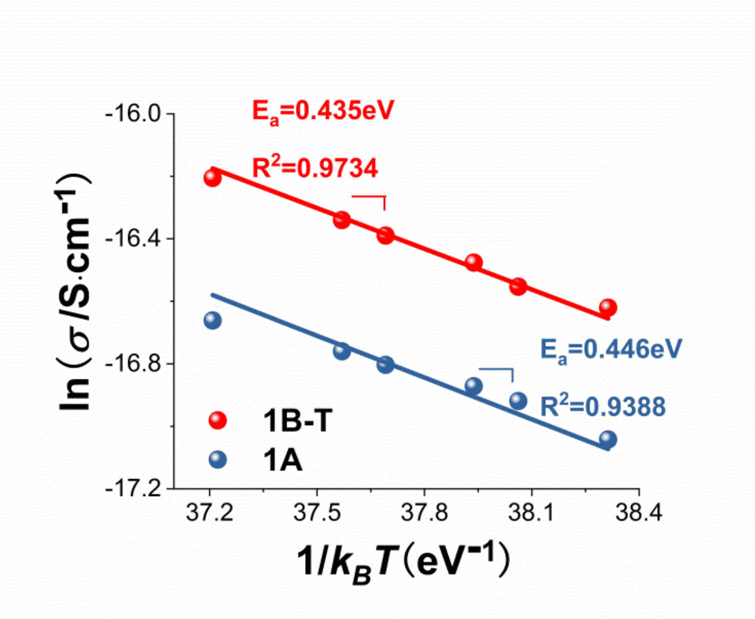

Supplementary Fig. 7 **Temperature-dependent conductivities of 1A and 1B-T**. $\ln\sigma = -E_a/k_B T$ + constant, where E_a is the activation energy, k_B is Boltzmann constant, T is temperature, and σ is conductivity.

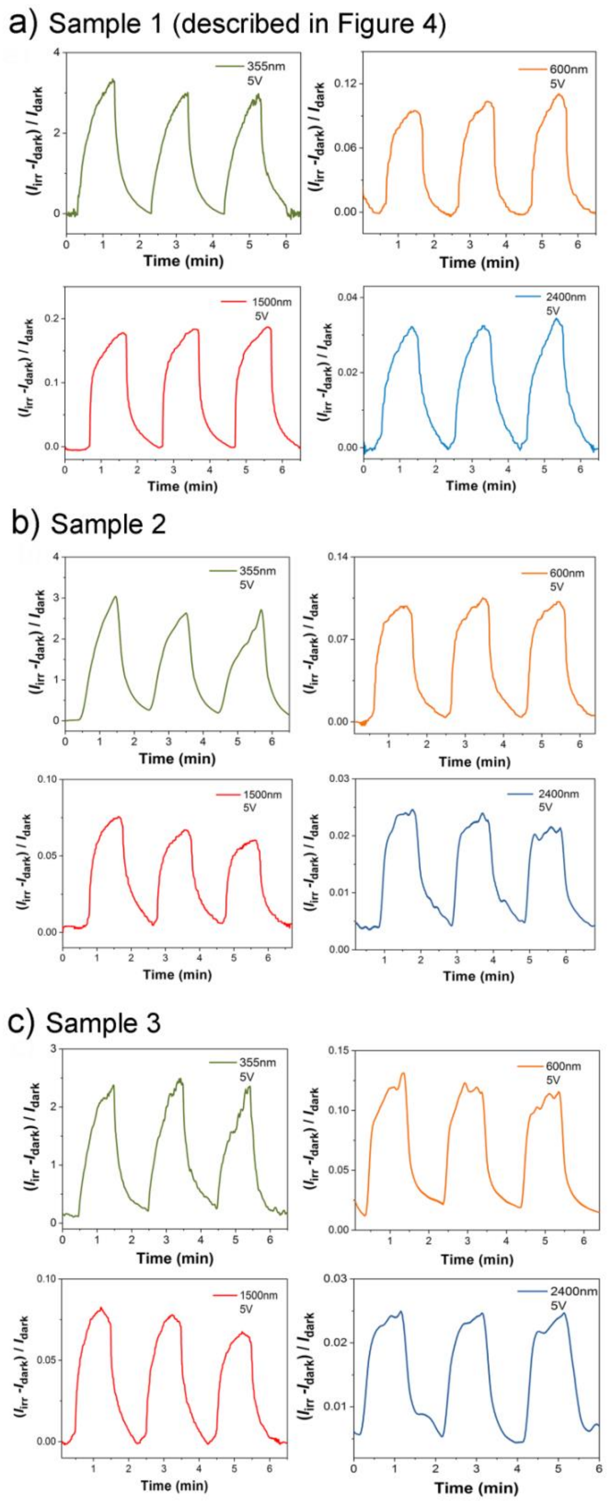

Supplementary Fig. 8 Photoswitching behavior for three samples of 1B-T. Power for each wavelength: a) 355 nm, 2.1 W; 600 nm, 5.0 mW; 1500 nm, 23 mW; 2400 nm, 6.0 mW; b) 355 nm, 2.1 W; 600 nm, 12 mW; 1500 nm, 12 mW; 2400 nm, 3.0 mW; c) 355 nm, 2.1 W; 600 nm, 16 mW; 1500 nm, 15 mW; 2400 nm, 3.5 mW.

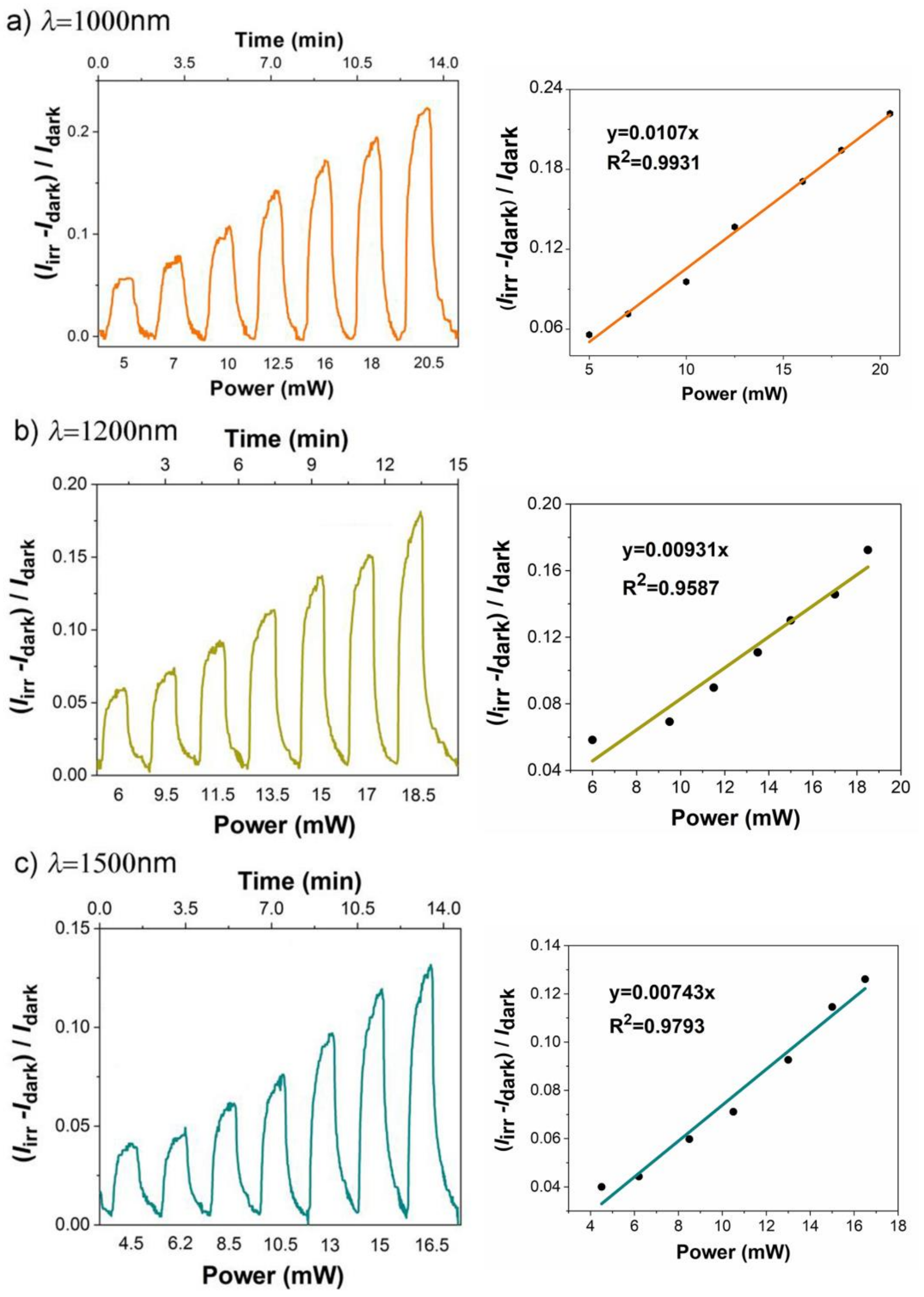

Supplementary Fig. 9 Plot of relative photocurrent gain versus power. Laser power-dependent photoresponse behavior of **1B-T** with 1000 nm (a), 1200 nm (b) and 1500 nm (c) lasers, respectively. As can be seen, photocurrent gain shows a linear correlation to power, which means that a higher power density produces more carriers in a specific optical power range.

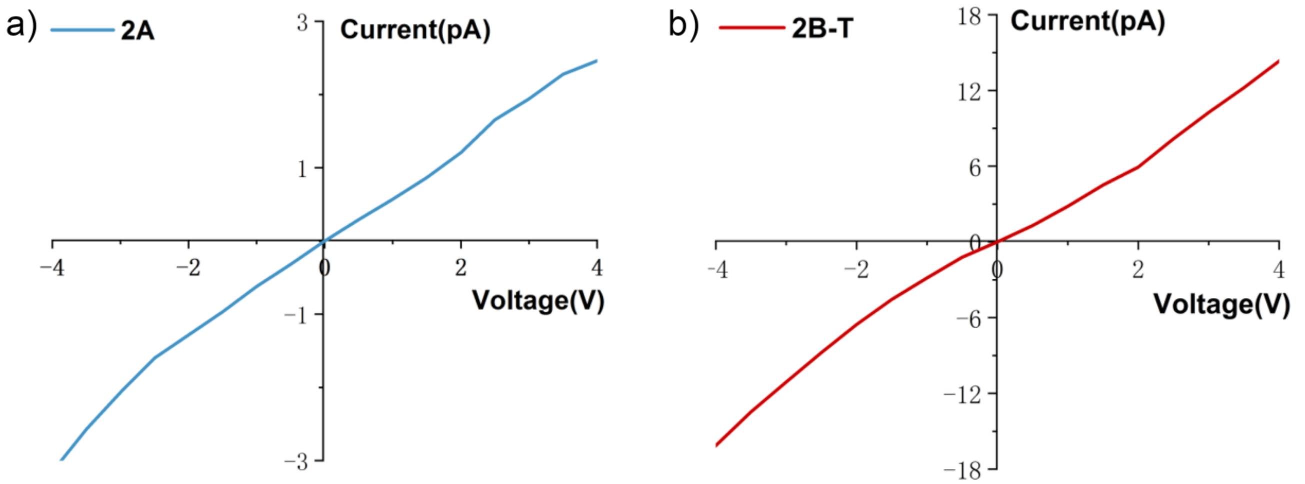

Supplementary Fig. 10 **Current–voltage (*I*–*V*) plots**. *I*-*V* plots featured Ohmic connect at 298 K in vacumn for **2A** (a) and **2B-T** (b).

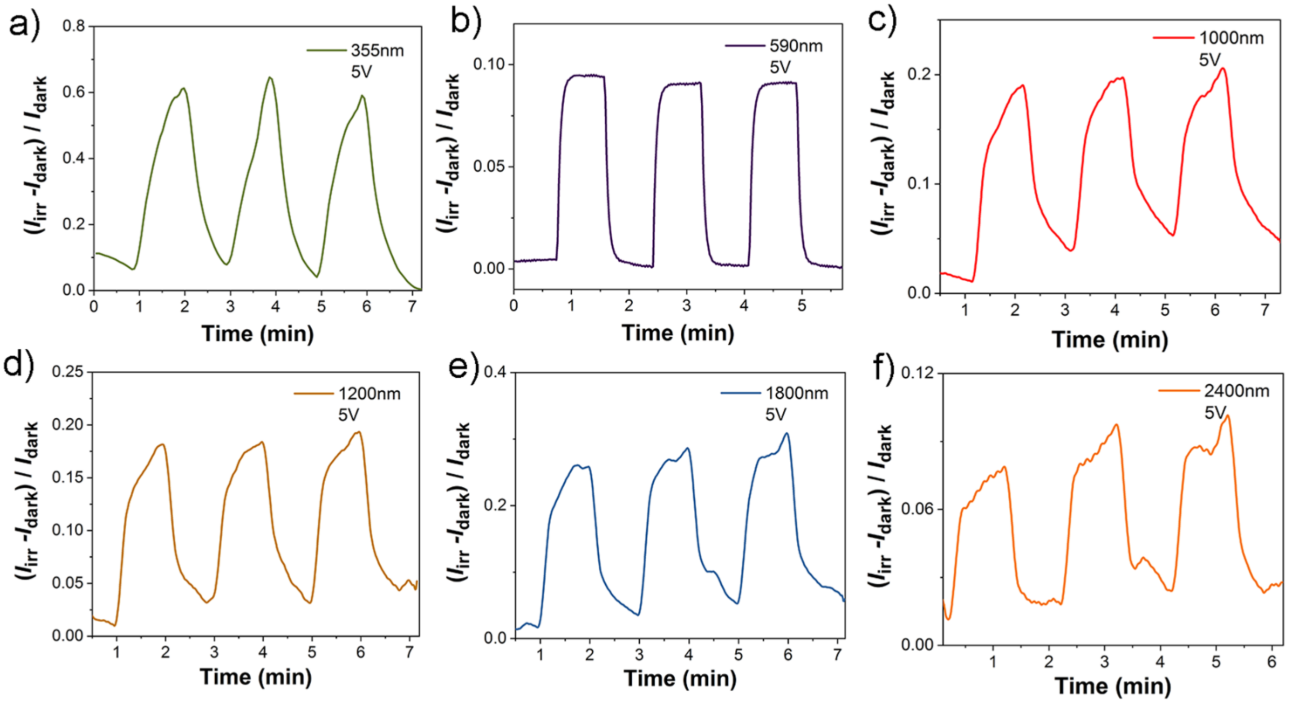

Supplementary Fig. 11 **Photoresponse behavior with different wavelengths of the 2B-T**. Power for each wavelength: a) 355 nm, 2.1 W; b) 590 nm, 19.2 mW; c) 1000 nm, 28 mW; d) 1200 nm, 25 mW; e) 1800 nm, 25 mW; f) 2400 nm, 7 mW.

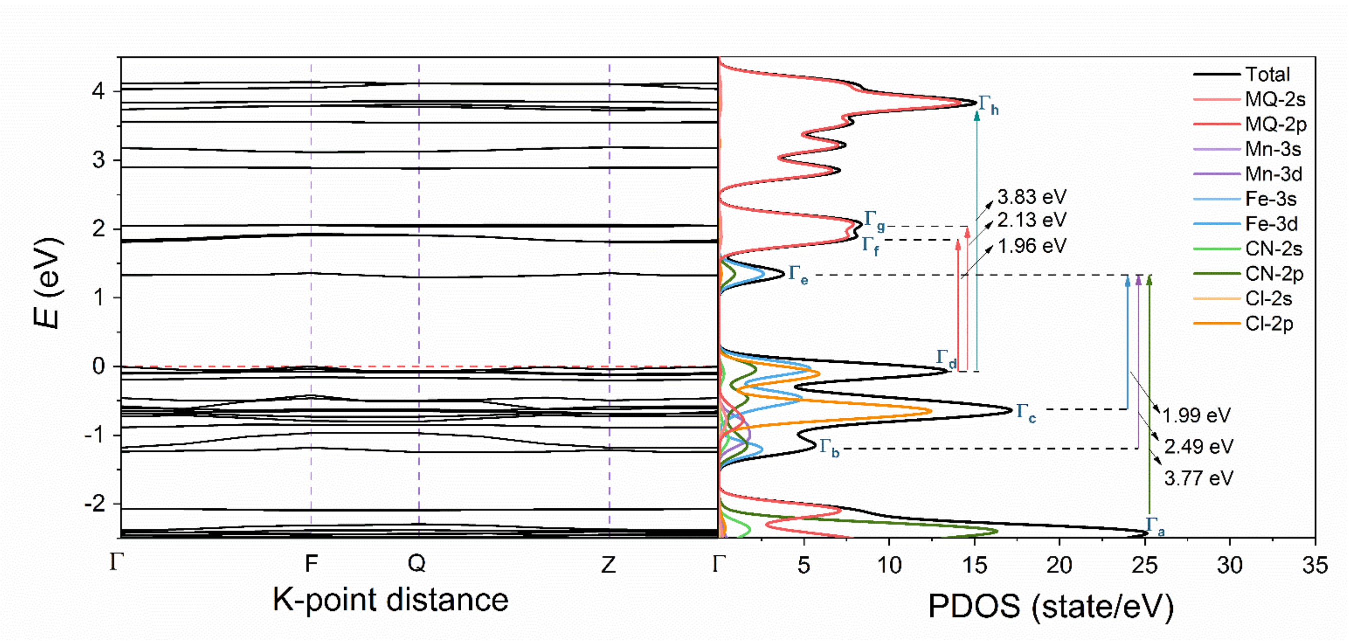

Supplementary Fig. 12 **Band structure and the density of states of 1**. Γ$_a$-Γ$_h$ denote the region maxima of the density of states. Γ$_a$ →Γ$_e$, Γ$_b$ →Γ$_e$, and Γ$_c$ →Γ$_e$ contain CN → Fe^{III} charge-transfer, d−d transition band of Fe^{III} and MMCT, respectively. Γ$_d$ →Γ$_f$, Γ$_d$ →Γ$_g$, and Γ$_d$ →Γ$_h$ can be assigned to Cl^-/CN^-/Fe^{III} → MQ^+. For the convenience of discussion, the up-spin bands and the down-spin bands are not given separately.

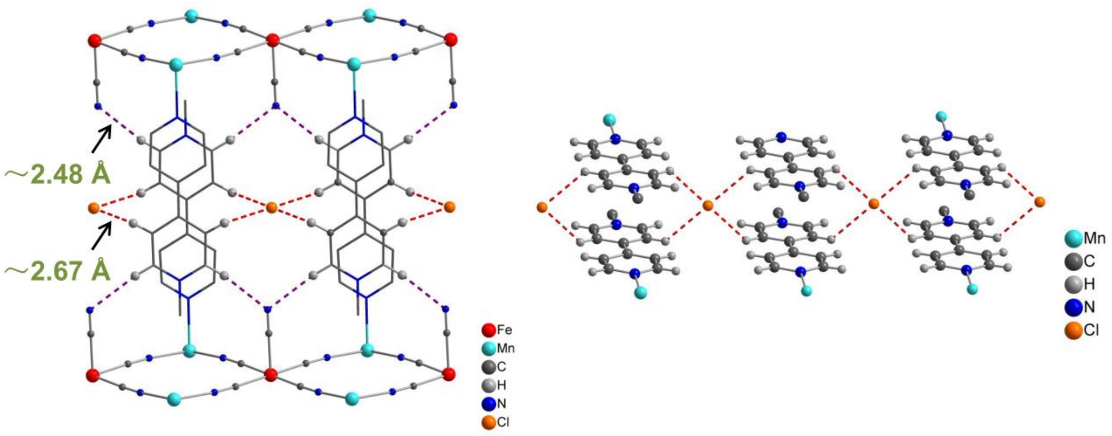

Supplementary Fig. 13 **Hydrogen-bonding diagram of 1**.

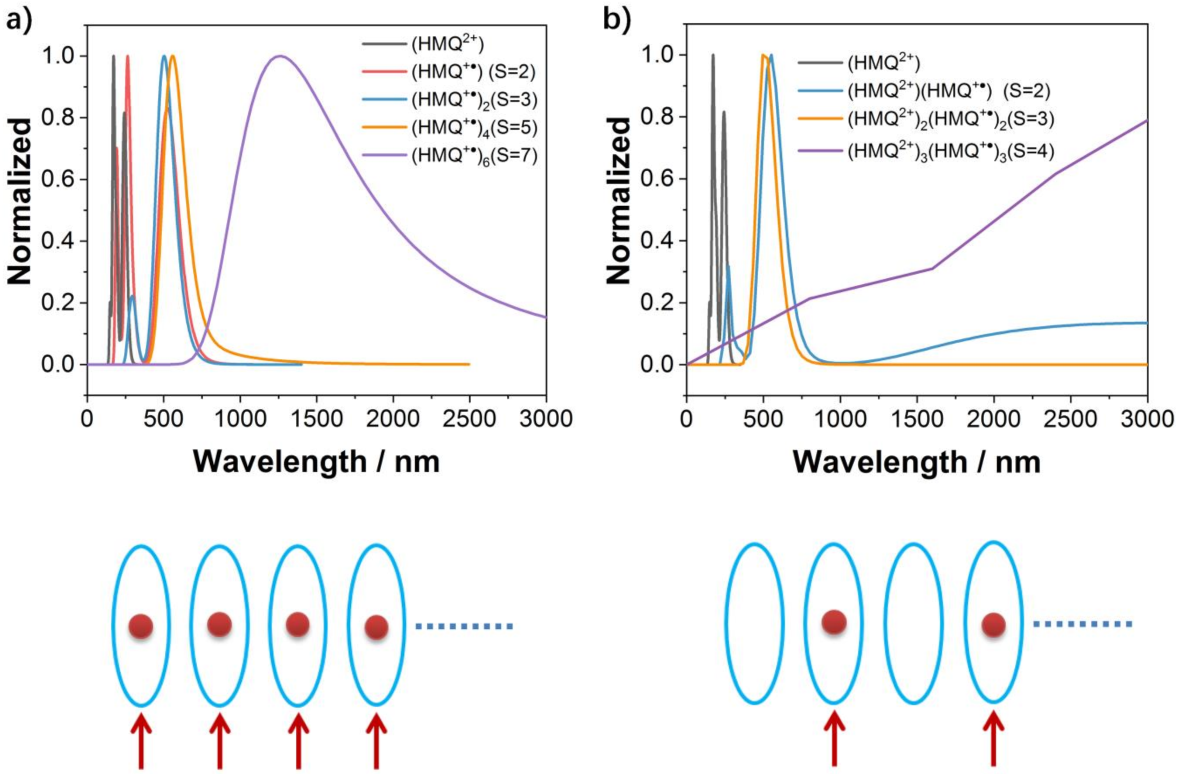

Supplementary Fig. 14 Calculations of electron absorption spectra. The π-stacked $[(HMQ^{2+})(HMQ^{+\bullet})]_n$ (a) and $[(HMQ^{+\bullet})(HMQ^{+\bullet})]_n$ (b) moieties (n refers the number). The models were truncated from the sing-crystal structure and modified using H atoms to replace metal atoms. HMQ^{2+} = *N*-protonated *N'*-methyl 4,4'-bipyridinium. $HMQ^{+\bullet}$ = one-electron reduced HMQ^{2+}. All calculations were performed at the pbe1pbe/6-31g* level using the TD-DFT method.

2 Supplementary Tables.

Supplementary table 1 Crystal and structure refinement data.

	**1** (as-synthesized)	**2** (as-synthesized)
**Formula**	$C_{28}H_{28}ClFeMnN_{10}O_3$	$C_{28}H_{28}ClFeZnN_{10}O_3$
M_r	698.83	709.27
**Crystal size** (mm^3)	0.2 × 0.16 × 0.13	0.18 × 0.13 ×0.08
**Crystal system**	Monoclinic	Monoclinic
**Space group**	*P*2/*m*	*P*2/*m*
a **(Å)**	7.332(4)	7.332(4)
b **(Å)**	7.688(3)	7.688(3)
c **(Å)**	13.528(7)	13.528(7)
β **(deg)**	98.064(14)	98.064(14)
V (Å^3)	755.0(6)	755.0(6)
D_{calcd} (g/cm^3)	1.510	1.533
Z	1	1
F**(000)**	358	363
**Abs coeff** (mm^{-1})	1.036	1.408
**Reflns collcd/unique** (R_{int})	8130/1886 (0.0617)	7142/1504 (0.0727)
**Data/params/restraints**	1886/121/0	1504/121/6
R_1[a]	0.1216	0.1167
ωR_2[b]	0.1569	0.2132
**GOF on** F^2	1.057	1.027
$\Delta\rho_{max}$ **and** $\Delta\rho_{min}$ (e/Å^3)	0.907 and –0.585	1.258 and –0.609

[a]$R_1 = \Sigma||F_o|-|F_c||/\Sigma|F_o|$, [b]$\omega R_2 = \{\Sigma\omega[(F_o)^2-(F_c)^2]^2/\Sigma\omega[(F_o)_2]^2\}^{1/2}$.

3 Supplementary Note 1:

Measurement of the relative spin density.

The relative spin density of **1** and **2** were measured using standard sample set in the instrument as a reference. The test conditions and methods are consistent with the standard sample. The following table shows the relative spin densities that are obtained by subtracting the recorded data to that of **1A** or **2A**. **1B-P/2B-P** are samples that were irradiated by an Xe lamp for 70 min; **1B-T/2B-T** are samples that were thermally annealed at 180 °C for 150 min.

Supplementary table 2 **Relative spin densities**.

Sample name	Relative spin density / mol^{-1}	Sample name	Relative spin density / mol^{-1}
**1B-P**	2.478×10^{21}	**2B-P**	1.020×10^{17}
**1B-T**	5.637×10^{22}	**2B-T**	1.303×10^{20}
